# Two green spectrofluorimetric methods for the assay of atomoxetine hydrochloride in pure form and commercial capsules with application to content uniformity testing

**DOI:** 10.1098/rsos.230010

**Published:** 2023-04-05

**Authors:** Neamat T. Barakat, Amina M. El-Brashy, Mona E. Fathy

**Affiliations:** Department of Pharmaceutical Analytical Chemistry, Faculty of Pharmacy, Mansoura University, Mansoura 35516, Egypt

**Keywords:** atomoxetine, spectrofluorimetry, SDS, Erythrosine B, content uniformity testing, green analysis

## Abstract

Atomoxetine hydrochloride (ATX) is a potent and non-stimulant drug which was approved for the treatment of attention-deficit hyperactivity disorder. Owing to its importance, two green, simple and validated spectrofluorimetric methods were developed for its sensitive assay in pure and capsule forms. The first method (Method I) relied on measuring the enhanced fluorescence of ATX by the use of sodium dodecyl sulfate in alkaline medium at *λ*_ex_ 227 nm/*λ*_em_ 298 nm. The second method (Method II) involved complex formation of ATX with Erythrosine B (EB) in aqueous acidic solution resulting in quantitative quenching of the EB naive fluorescence. This complex was formed in the presence of Britton–Robinson buffer (pH 4.0). The difference in fluorescence intensity was measured at *λ*_ex_ 527/*λ*_em_550 nm. The calibration curves were linear through the ranges of 0.2–2.0 µg ml^−1^ for Method I, 0.2–4.0 µg ml^−1^ for Method II with good correlation coefficient (*r* = 0.9998) for both methods. The suggested methods were perfectly applied for determination of ATX in its commercial capsules and content uniformity test. The greenness of the proposed methods was confirmed by three different assessment tools and it was found that both methods were green, eco-friendly and environmentally safe.

## 1. Introduction

**Figure 1.**
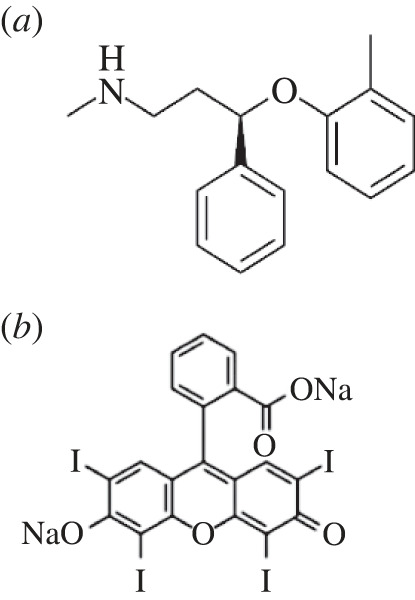
Chemical structure of ATX (*a*) and EB (*b*).

Atomoxetine HCl (figure 1*a*) is chemically defined as (3R)-N-Methyl-3-(2-methylphenoxy)-3-phenylpropan-1-amine hydrochloride [1]. It is a potent and selective norepinephrine reuptake inhibitor used to treat attention-deficit hyperactivity disorder in children, adolescents and adults [2].

ATX is official in the United States Pharmacopeia (USP) [3] as well as in the British Pharmacopeia (BP) [4]. USP recommended an HPLC method for the assay of ATX using C8 column and acetonitrile/phosphate buffer in the ratio (38 : 62 V/V) as a mobile phase with UV detection at 220 nm [3]. BP also recommended a liquid chromatographic method using end capped octylsilyl silica gel column with UV detection at 215 nm. The mobile phase used for separation is a mixture of propanol and 0.059% sodium octanesulfonate solution in phosphoric acid (pH 2.5) in the ratio (27:73 V/V) [4].

Literature shows that there are many analytical methods for the assay of ATX in different matrices as spectrofluorimetric [5,6], spectrophotometric [5,7,8], HPLC [9–12], electrochemical [13,14], capillary electrophoretic [15] and LC-MS [16–19] methods.

Most of the reported methods relied on chromatographic techniques which suffered from many disadvantages such as expensive instruments, higher quantities of hazardous solvents required, the need for a highly trained person, and a tedious and time-consuming sample pre-treatment process. On the other side, the spectroscopic methods are simpler and rapid with low cost. In addition, spectrofluorimetric methods have the merits of being more sensitive and selective. The previously reported spectrofluorimetric methods are based on the drug reactions with either 4-Chloro-7-nitrobenzo-2-oxa-1,3-diazole (NBD-CL) in alkaline medium [5] or eosin in acidic medium [6].

Although the reported method [5] is more sensitive (10.0–500.0 ng ml^−1^), the proposed methods are more rapid, simpler and do not require heating or time for the reaction to proceed. Moreover, the suggested methods are greener because they use a green solvent (water) while the reported method [5] used a non-green and hazardous organic solvent (methanol).

Reviewing the literature showed that no spectrofluorimetric method has been published about the native fluorescence of ATX. In addition, none of the previously published fluorimetric methods [5,6] include assessment of a method's greenness.

The proposed techniques aimed to develop two spectrofluorimetric methods for sensitive and selective determination of ATX. The proposed methods depended on either the enhanced native fluorescence of ATX in alkaline organized medium or the formation of ion-pair complex with Erythrosine B (EB) in acidic medium. These proposed methods were efficaciously applied to the assay of ATX in pure form and commercial capsules. In addition, the content uniformity testing and assessment of method greenness were performed. Furthermore, the limiting logarithmic method and stern volmer plots were used to explain the mechanism of formation of ATX-EB complex.

## 2. Practical

### 2.1. Instrumentation

Fluorescence intensity (FI) was measured using a fluorescence spectrophotometer (Agilent Cary Eclipse, USA) and the sensitivity was adjusted at 800 for Method I and 650 for Method II. Standard quinine sulfate solution was used to check the calibration and linearity of the device.

A Jenway 3510 pH meter was used for adjusting pH (UK).

A water bath from England Cambridge Ltd (Shaker) was used to monitor the temperature.

### 2.2. Materials and reagents

HPLC grade solvents and analytical reagent grade chemicals were used.

Atomoxetine hydrochloride (ATX) was kindly obtained as a gift from Multi-Apex Pharma S.A.E, (Badr City, Cairo, Egypt) with a purity of 99.93 ± 1.84% as calculated by the comparison method [4].

Atomox Apex® capsules claimed to contain 10 mg and 40 mg with batch no. 25 960 and 25 963, respectively (Multi-Apex Pharma S.A.E, Badr City, Cairo, Egypt).

Aqueous solution (1.7 × 10^−4^ M) of Erythrosine B was prepared (Acros-organics, Belgium).

Acetonitrile, methanol and ethanol (Fisher Scientific UK, Loughborough, Leics (UK)).

Sodium dodecyl sulfate (SDS), tween-80, β-cyclodextrin, phosphoric acid, acetic acid, hydrochloric acid, boric acid and sodium hydroxide were bought from El-Gomhouria Company (Mansoura, Egypt), but cetrimide was obtained from Winlab (UK).

1.0% (w/v) aqueous solutions of all organized media were prepared.

### 2.3. Buffer solutions

Britton–Robinson buffer (BRB) solution was prepared over a pH range 2.0–12.0 by mixing equimolar (0.04 M) of boric acid, acetic acid and phosphoric acid with equal volumes. Then, 0.2 M NaOH solution was used to adjust the pH. [20]

Borate buffer with pH range 6.0–10.0 was obtained by adding the appropriate volume of 0.2 M of sodium hydroxide to 0.2 M boric acid.

### 2.4. Standard solution preparation

0.02 g of ATX was dissolved in distilled water to prepare an aqueous stock solution (200 µg ml^−1^) for both methods (I and II). Then standard working solutions were prepared by further dilution of the stock solution with distilled water.

The solution was stored in a refrigerator at 2–8°C and was stable for at least 14 days.

### 2.5. Procedures

#### 2.5.1. Construction of calibration curves

##### 2.5.1.1. Method I

Various volumes of ATX aqueous solution were added to a series of volumetric flasks (10 ml), then 1.3 ml of 1 M NaOH and 0.7 ml of 1% SDS solution were added. The resulting solutions were then completed with distilled water to the mark and mixed well. The relative fluorescence intensities (RFIs) of the ATX final solutions were instantaneously measured at *λ*_ex_ 227 nm / *λ*_em_ 298 nm parallel with blank measurement. Then a calibration curve was constructed by plotting RFI values against drug concentrations to obtain the corresponding regression equation.

##### 2.5.1.2. Method II

Various volumes of ATX aqueous solution were added to a set of volumetric flasks (10 ml), then 1.0 ml of BRB solution (pH 4.0) and 0.6 ml of 1.7 × 10^−4^ M EB solution were added. Flasks were completed to the volume with distilled water and mixed well. The difference in FI of EB before and after the addition of ATX (ΔF) was immediately measured at 550 nm after being excited at 527 nm. The study was performed simultaneously against a blank measurement. *Δ*F values were plotted against ATX final concentration (μg ml^−1^) to obtain the desired calibration curve where the corresponding regression equation can be obtained.

#### 2.5.2. Analysis of atomoxetine hydrochloride in Atomox Apex® capsules 40 mg

Ten capsules were weighed, evacuated and their contents were mixed well. A quantity equivalent to 20.0 mg of ATX was taken from the mixed powder and added to a 100 ml volumetric flask, then completed to mark with distilled water, sonicated for about 45 min and filtered. Next, procedures under §2.5.1. were performed as illustrated before. From pre-constructed calibration curves for both methods, the correlative regression equations were used to obtain ATX concentration in its pharmaceutical dosage form.

#### 2.5.3. Content uniformity testing

Ten capsules of Atomox Apex® 10 mg were evacuated separately and each capsule content was added to a volumetric flask (100 ml) then completed with distilled water to the mark, sonicated for 45 min and filtered. The procedures in §2.5.1 for both methods to determine ATX concentration in its commercial capsules were then applied and each capsule % found was obtained from the previously established calibration curves for both methods. The test was performed according to the United States Pharmacopeial Guidelines [21].

## 3. Results and discussion

**Figure 2.**
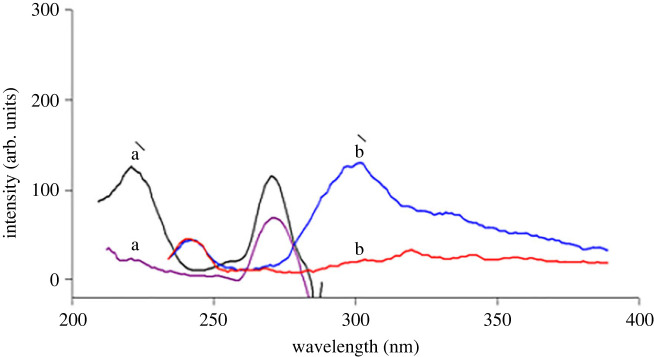
Excitation and emission fluorescence spectra of (a**,**b) blank (water). (a′, b′) native fluorescence of ATX (2.0 µg ml^−1^) in water.

**Figure 3.**
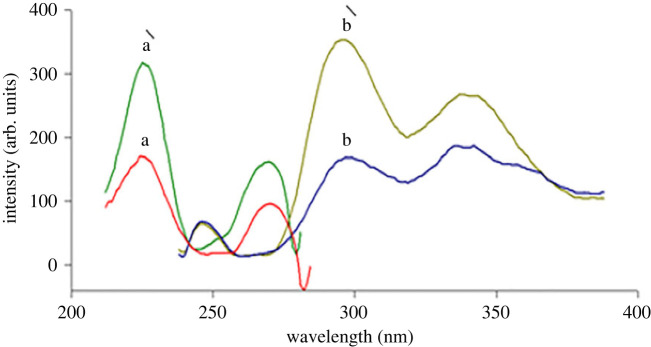
Excitation and emission fluorescence spectra of (a**,**b) blank alkaline SDS solution (1.3 ml of 1 M NaOH + 0.7 ml of 1% SDS). (a′, b′) ATX (0.9 µg ml^−1^) in alkaline SDS solution (1.3 ml of 1 M NaOH + 0.7 ml of 1% SDS).

ATX has a relatively low intrinsic fluorescence in water at *λ*_ex_ 222/*λ*_em_301 nm, as shown in figure 2. So, two methods have been described for improving its sensitivity. The first method is based on using an ionic surfactant (SDS) solution in alkaline medium which leads to greatly enhanced ATX fluorescence (350%). This enhanced FI was measured at 298 nm after being excited at 227 nm as indicated in figure 3. This suggested procedure led to the development of a highly sensitive technique for estimation of ATX in its pure and pharmaceutical dosage form.

**Figure 4.**
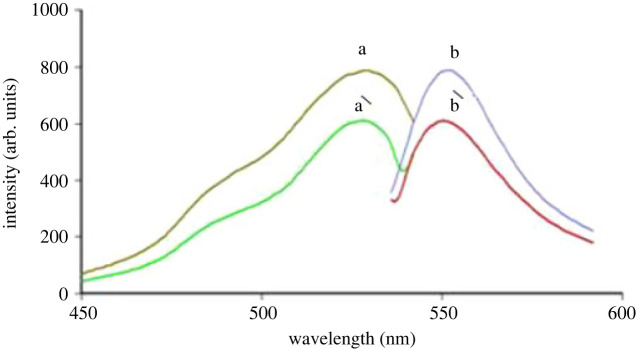
Excitation and emission fluorescence spectra of (a,b) blank EB (1.7 × 10^−4^ M) in BRB (pH 4.0). (a′, b′) ATX (2.0 µg ml^−1^) complex with EB(1.7 × 10^−4^ M).

The fluorescence quenching technique is another method that was adopted to increase the sensitivity of the ATX analysis in pure form and capsules. It is based on the fact that the EB aqueous solution exhibits a very high native FI in acidic medium at 554 nm when excited at 527 nm. After addition of ATX to EB in BRB solution (pH 4.0), a non-fluorescent ion-pair was formed producing a remarkable decrease in the EB FI as in figure 4.

EB (figure 1*b*) was previously used for estimation of compounds of pharmaceutical interest such as flubendazole and mebendazole [22], tamoxifen citrate and clomiphene citrate [23] and duloxetine [24]. It is an acidic dye which has a free phenolic hydroxyl group that more readily ionized in slightly acidic conditions (pH 4.0). Under the same acidic conditions, the cationic form of ATX is predominant because of the ionization of its secondary amino group. Then, two compounds form a complex via electrostatic interaction between the two oppositely charged ions.

### 3.1. Experimental conditions optimization

Different parameters affecting both suggested methods were then studied and optimized.

#### 3.1.1. Method I

##### 3.1.1.1. Effect of organized media

RFI of ATX was examined using different organized media like SDS, tween 80, cetrimide and β-cyclodextrin. It was found that cetrimide had no effect, while β-cyclodextrin and tween 80 decreased RFI of ATX. However, RFI was enhanced greatly in the presence of SDS. Therefore, SDS was used in this study.

##### 3.1.1.2. Effect of 1% sodium dodecyl sulfate volume

**Figure 5.**
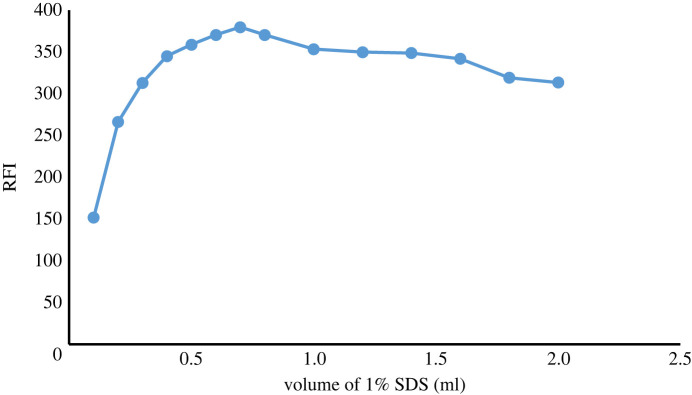
Effect of volume of (1.0% w/v) SDS solution on native FI of ATX (2.0 µg ml^−1^).

Different volumes of 1% SDS (0.1–2 ml) were examined and it was found that 0.7 ml was the best volume (figure 5).

##### 3.1.1.3. Effect of pH

To study this effect, 0.2 M borate buffer of pH range 6.0–10.0, 0.2 M BRB buffer of pH range 2.0–12.0, 1 M NaOH and 1 M HCl were tried. It was found that using the buffer media and 1 M HCl had no significant effect. However, the RFI of ATX was increased by using 1 M NaOH solution. Hence, it was used during this study.

##### 3.1.1.4. The effect of volume of 1 M NaOH

**Figure 6.**
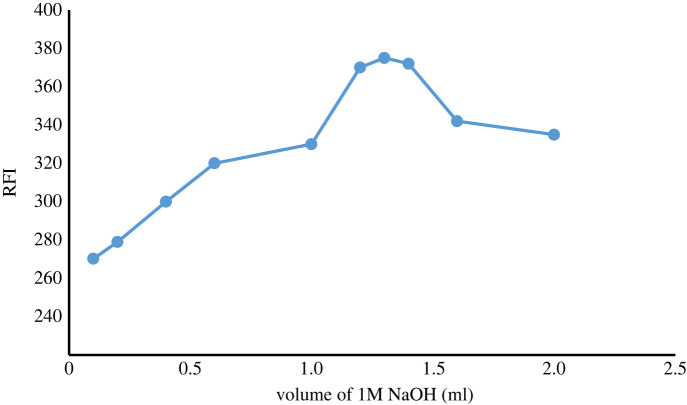
Effect of volume of 1 M NaOH on FI of ATX (2.0 µg ml^−1^).

Different volumes of 1 M NaOH were tried and it was found that 1.3 ml achieved the optimal RFI of ATX (figure 6)

##### 3.1.1.5. The effect of different diluting solvents

**Figure 7.**
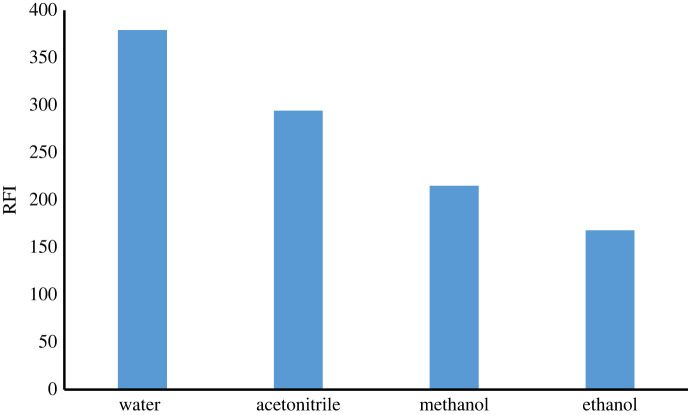
Effect of different diluting solvents on FI of ATX (2.0 µg ml^−1^).

Different solvents like methanol, distilled water, ethanol and acetonitrile were tried. It was found that the greatest RFI value was obtained by using distilled water as a diluting solvent during this work. (figure 7)

#### 3.1.2. Method II

##### 3.1.2.1. The effect of Britton–Robinson buffer solution pH and volume

**Figure 8.**
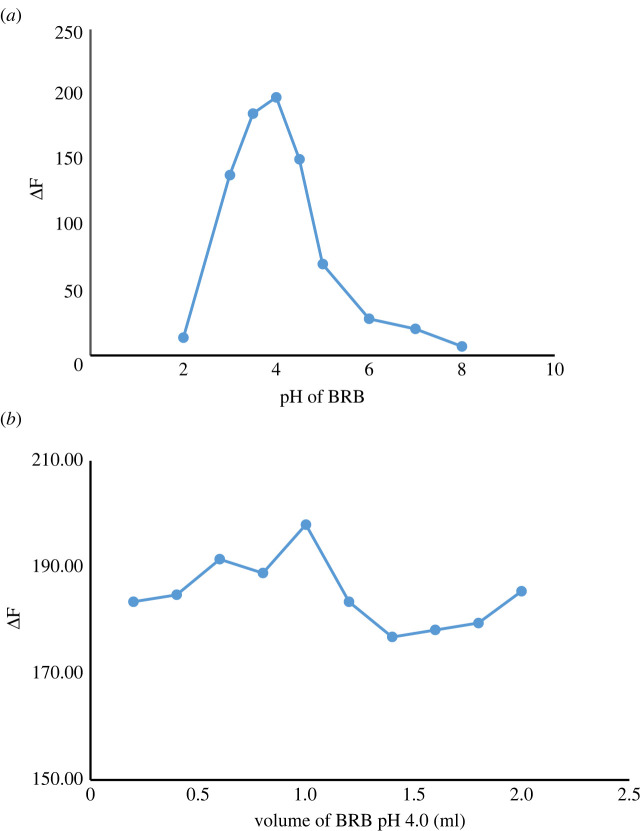
(*a*) Effect of pH of BRB on the fluorescence quenching of EB by ATX (2.0 µg ml^−1^). (*b*) Effect of volume of BRB (pH 4.0) on the fluorescence quenching of EB by ATX (2.0 µg ml).

The formation and stability of ATX-EB complex were significantly influenced by the pH of BRB solution. So, different pH values (2.0–8.0) were tried (figure 8*a*). The maximum *Δ*F value was observed by using pH 4.0. It has been observed that decreasing or increasing the pH values from 4.0 results in decreasing *Δ*F values. Consequently, BRB of pH 4.0 ± 0.1 was used in this work to obtain the greatest sensitivity.

The effect of BRB volume was studied in the range of 0.2–2 ml. It was found that ΔF values slightly increased by increasing the BRB volume to 1 ml, and after that no more increase or even decrease was obtained. Consequently, 1.0 ml of BRB (pH 4.0) was the best choice for ATX-EB ion-pair formation (figure 8*b*).

##### 3.1.2.2. The effect of Erythrosine B volume

**Figure 9.**
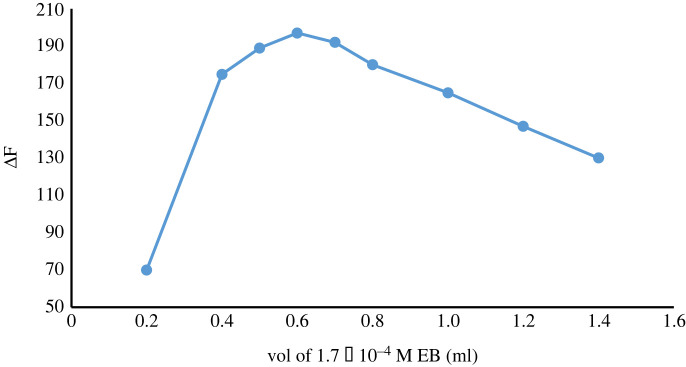
Effect of volume of EB (1.7 × 10^−4^ M) on the complex formation with ATX (2.0 µg ml^−1^).

The effect of volume of 1.7 × 10^−4^ M EB on ΔF values was observed over the range 0.2–1.4 ml. The greatest ΔF values were obtained by using 0.6 ± 0.1 ml of EB (figure 9).

##### 3.1.2.3. The effect of organized media

For the sake of optimizing the ATX quenching effect on the native FI of EB, different organized media, such as SDS, tween 80, cetrimide and β-cyclodextrin were tried. It was found that no positive effect on ΔF values was obtained by using these organized media. Therefore, none of them were used in this work.

##### 3.1.2.4. The effect of different diluting solvent

To select the most suitable solvent for the reaction between ATX and EB, different solvents, such as ethanol, methanol, acetonitrile and distilled water were tried. It was found that the highest ΔF values were observed by using distilled water. So, it was the best solvent of choice throughout this study.

##### 3.1.2.5. The effect of time on atomoxetine hydrochloride–Erythrosine B complex formation and stability

The reaction between ATX and EB was studied at different periods. It was found that the reaction was instantaneous and the resulting complex was stable for about 100 min.

### 3.2. Atomoxetine hydrochloride and Erythrosine B reaction mechanism

#### 3.2.1. The stoichiometry of the reaction between atomoxetine hydrochloride and Erythrosine B

**Figure 10.**
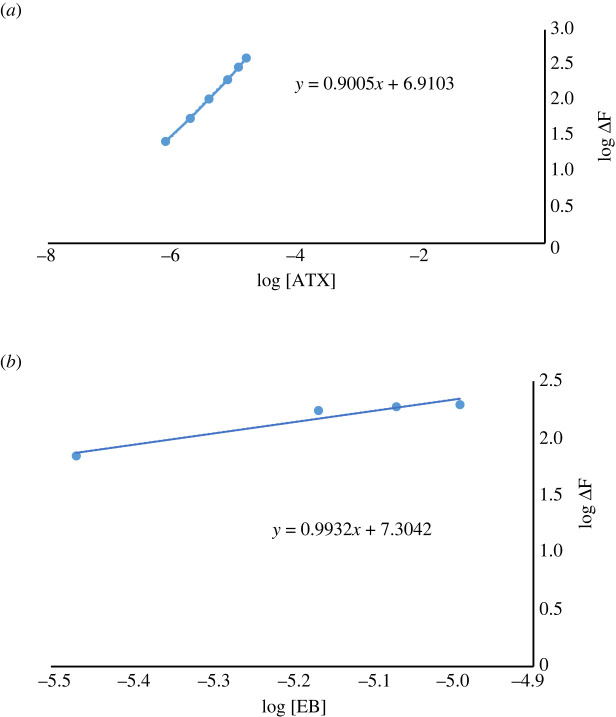
Stoichiometry of the reaction between ATX with EB using limiting logarithmic method.

The molar ratio was attained by applying the limiting logarithmic method [25]. By plotting log ΔF values against log[ATX] and log ΔF versus log[EB], two straight lines were obtained, and their slopes were calculated and found to be 0.90 and 0.99, respectively. (figure 10*a,b*).

**Scheme 1.**
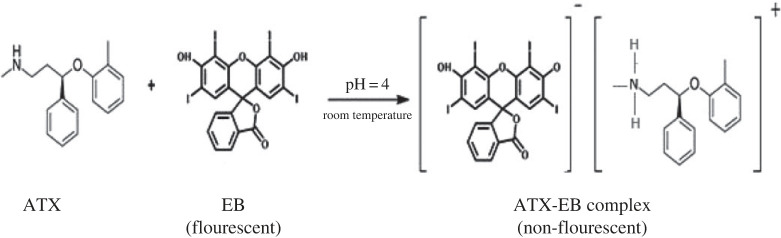
The reaction mechanism for complex formation between ATX and EB.

This proved a 1 : 1 molar reactivity. This ratio was explained by the presence of one amino group in ATX. The ATX positively charged amino group reacted with the EB negatively charged hydroxyl group by electrostatic interactions, leading to the ion-pair complex formation. Based on the obtained molar ratio, the reaction mechanism between ATX and EB was proposed in scheme 1.

#### 3.2.2. The mechanism of quenching of the ion-pair between atomoxetine hydrochloride and Erythrosine B

There are various examples of molecular interactions that cause quenching of fluorescence, [26] such as molecular rearrangement, static, dynamic quenching, excited-state and energy transfer reactions. Consequently, the Stern–Volmer method [27,28] was applied to determine quenching type in the suggested EB–ATX interaction. The Stern–Volmer plots were obtained by plotting F°/F versus [Q] according to the equation:

$$\frac{F{\;}^{\circ }}{F}=1+K_{\mathrm{SV}}[Q],$$

where K_SV_ is the Stern–Volmer quenching constant, [Q] is the molar concentration of ATX, F and F^0^ are the EB fluorescence intensities with and without the addition of ATX, respectively.

**Figure 11.**
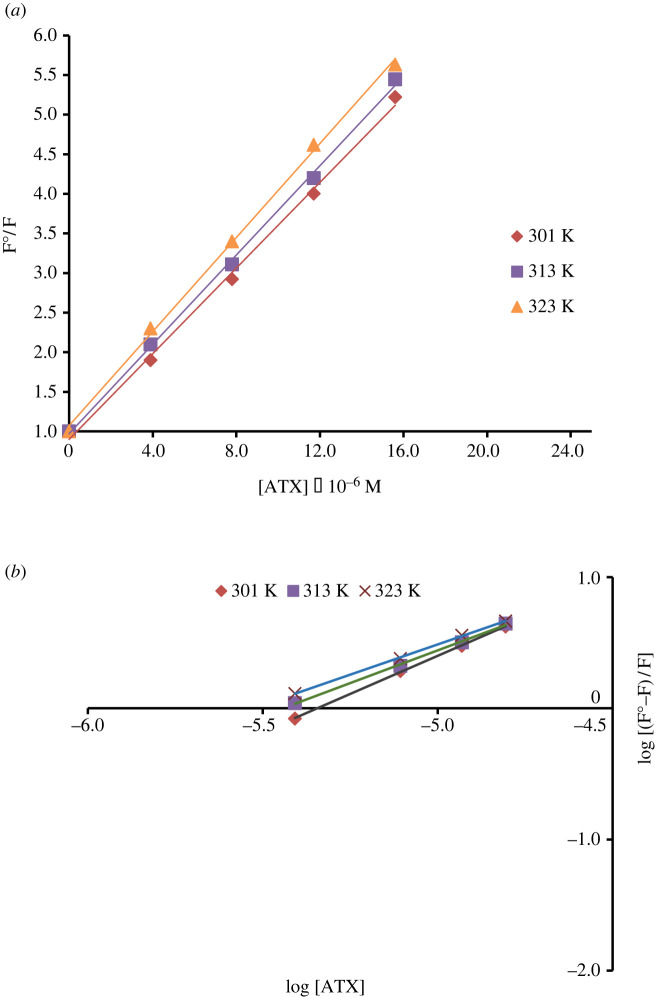
(*a*) Stern–Volmer plot for fluorescence quenching at 303 K, 313 K and 323 K. (*b*) Modified Stern–Volmer plot for fluorescence quenching at 303 K, 313 K and 323 K.

It was found from the linear relationship (*r* = 0.99, 0.99 and 0.99) between F^0^/F and [Q] that there is only one type of quenching process (static or dynamic) as shown in (figure 11*a*).

*Δ*F were measured at room and high temperatures (301–313–323)°K to determine the type of quenching process and by constructing Stern–Volmer plots at these raised temperatures, it was shown that K_SV_ increased at elevated temperature indicating a dynamic quenching behaviour [27–29] (table 1).

**Table 1.** Summary of the Stern–Volmer method for the reaction of ATX with EB.

temperature (°K)	Stern–Volmer quenching constant (KSV) × 10^6^ (l mol^−1^)	correlation coefficient (r)	bimolecular quenching constant (Kq) × 10^15^ (L mol^−1^ S)
301	0.2696	0.99	3.03
313	0.2809	0.99	3.16
323	0.2961	0.99	3.33

Moreover, the bimolecular quenching constant (Kq), which is an indicator of the efficiency of the fluorescence and accessibility of the EB (fluorophore) to ATX (the quencher), can be calculated, according to equation [29]:

$$Kq=\frac{K_{\mathrm{SV}}}{\tau_{0}},$$

where K_SV_ is the Stern–Volmer quenching constant and ***τ*****_0_** is the native radiation lifetime of EB (89 ps) [30]. Kq is the bimolecular quenching constant. It was found that the calculated values of Kq were 3.03 × 10^15^, 3.16 × 10^15^ and 3.33 × 10^15^ l mol^−1^ S^−1^ at (301–313–323)°K, respectively.

#### 3.2.3. Calculation of the rate constants (Kb), binding sites and free energy changes (*Δ*G°)

By using the modified Stern–Volmer plot (figure 11*b*), the number of binding sites (*n*) and rate constant of the reaction between ATX and EB (Kb) can be calculated from the following equation [31,32]:

$$Log\left(\frac{\left(F{\;}^{\circ }-F\right)}{F}\right)=LogKb+nLog[D],$$

where Kb is the reaction rate constant, *n* is the binding sites number, [D] is the ATX molar concentration, F^0^ and F are the FI of EB in the absence and presence of ATX, respectively. Kb was calculated and found to be 16.74 × 10^5^ l mol^−1^ and the number of binding sites was approximately one. This confirms the molar ratio (1 : 1) resulting from the limiting logarithmic method as shown in table 2.

**Table 2.** Binding sites, rate constants and free energy changes.

temperature (°K)	reaction rate constant (Kb) (×10^5^ l mol^−1^)	*n*	Gibb's free energy (*Δ*G) (kJ mol^−1^)
301	16.7492	1.1647	−35.616
313	2.7980	1.000	−33.216
323	1.2711	.9234	−31.215

Using the Kb value within the following equation to calculate Gibb's free energy (ΔG°):

$${\Delta G}^{\circ }=-RTlnKb,$$

where Kb is the reaction rate constant, *R* (8.314 J K^−1^ mol^−1^) is the universal gas constant and *T* is the temperature in Kelvin.

ΔG° was calculated and its value was −35.61 kJ mol^−1^ (table 2).

Furthermore, this negative value indicates the spontaneity and feasibility of the reaction at ambient temperature.

### 3.3. Validation of the two spectrofluorimetric methods

Both methods were validated according to (ICH) Guidelines [33]. All parameters of validation including linearity, range, accuracy, precision, specificity, limit of detection and limit of quantitation were studied.

#### 3.3.1. Linearity and concentration range

For Method I: the constructed calibration curve shows a linear dependence of RFI values on ATX concentrations throughout the specified range (0.2–2.0 µg ml^−1^). The derived regression equation was

RFI=14.58+183.98C.

For Method II: The calibration curve was obtained by plotting (ΔF) values versus corresponding concentrations of the ATX, linearity was achieved within the range 0.2–4.0 µg ml^−1^ and the resulting regression equation was as follows:

ΔF=7.16+97.63C.

Correlation coefficients (r), intercepts (a) and slopes (b) for the calibration data for both methods are elucidated in table 3.

**Table 3.** Performance data for the determination of ATX using the proposed methods.

parameters	Method I	Method II
linearity range (µg ml^−1^)	0.2–2.0	0.2–4.0
intercept (a)	14.58	7.16
slope (b)	183.98	97.62
correlation coefficient (*r*)	0.9998	0.9998
s.d. of residuals (*S_y/x_*)	2.93	2.83
s.d. of intercept (*S_a_*)	2.28	1.90
s.d. of slope (*S_b_*)	1.88	0.84
s.d.	1.28	1.45
% RSD	1.28	1.44
% error	0.52	0.59
LOD (µg ml^−1^)	0.04	0.06
LOQ (µg ml^−1^)	0.12	0.19

High correlation coefficients proved the excellent linearity [34] of the suggested methods. Moreover, the small values of the intercept s.d. (*S*_a_), slope standard deviation (*S*_b_) and residual standard deviation (*S*_y/x_) indicated small degree scattering of the points around the calibration curves (table 3).

#### 3.3.2. Accuracy

Six different concentrations through the specified range were analysed three times. The obtained results showed high degree of closeness between the true and measured values. In addition, the obtained values from Student's *t*-test and variance ratio F-test of both methods were lower than the tabulated ones [34], indicating that there was no significant difference between the two suggested methods and the comparison method. The comparison method described the spectrofluorimetric technique using eosin in acidic medium [6]. These obtained results show the good accuracy of both suggested methods (table 4).

**Table 4.** Assay results for the determination of ATX in the pure form using proposed and comparison methods. N.B the tabulated *t*- and *F*-values at *p* = 0.05 are 2.31 and 5.41, respectively [34].

parameter	Method I		Method II		comparison method [6]
	conc. taken (µg ml^−1^)	% found^a^	conc. taken (µg ml^−1^)	% found^a^	% found^a^
	0.2	100.00	0.2	101.00	98.40
	0.4	98.25	0.5	101.80	100.40
	0.8	99.13	1.0	101.60	102.30
	1.2	102.00	2.0	98.05	98.60
	1.6	100.31	3.0	99.30	
	2.0	99.30	4.0	100.73	
mean		99.83		100.41	99.93
s.d.		1.28		1.45	1.84
*t-test*		0.10		0.47	
*F-test*		2.04		1.63	

^a^Each result is the mean of three separate determinations.

#### 3.3.3. Precision

Intra-day precision (repeatability) and inter-day (intermediate) precision for both methods were determined by analysing three different concentrations three times in the same day and over three successive days (table 5).

**Table 5.** Precision data for the determination of ATX using the proposed methods.

method	conc. (µg ml^−1^)	intra-day precision			inter-day precision		
		mean ± s.d.	% RSD	% error	mean ± s.d.	% RSD	% error
Method I	0.4	98.62 ± 1.05	1.06	0.61	97.72 ± 0.68	0.69	0.40
	1.2	101.19 ± 0.46	0.45	0.26	101.87 ± 0.22	0.22	0.13
	2.0	99.10 ± 0.29	0.30	0.17	98.63 ± 0.14	0.14	0.08
Method II	1.0	100.21 ± 1.03	1.02	0.59	101.06 ± 0.78	0.77	0.45
	2.0	97.73 ± 0.52	0.53	0.30	97.99 ± 0.26	0.27	0.15
	3.0	99.30 ± 0.34	0.34	0.19	99.47 ± 0.17	0.17	0.10

The good repeatability and reliability of both suggested methods were confirmed by very low % RSD values.

#### 3.3.4. Sensitivity

The limits of detection (LOD = 3.3 *S*_a_/*b*) and limits of quantification (LOQ = 10 *S*_a_/*b*) were calculated (table 3) according to ICH guidelines [33], where *S*_a_ is the intercept standard deviation and *b* is the slope of the calibration curve.

The small values of LOD and LOQ for both methods confirmed the good sensitivity of both methods.

#### 3.3.5. Robustness

For evaluation of the robustness of both developed methods, minor modifications in the experimental parameters were carried out, such as 1% SDS volume (0.7 ± 0.1 ml), 1 M NaOH volume (1.3 ± 0.1 ml) for Method I and 0.2 M BRB pH (4.0 ± 0.1) and 1.7 × 10^−4^ M EB volume (0.6 ± 0.1 ml) for Method II.

It was observed that the resulting values were nearly constant with these minor modifications, which confirmed the robustness of the two proposed methods (table 6).

**Table 6.** Robustness of the suggested techniques for ATX.

parameter	mean ± s.d.	% RSD
Method I		
1% SDS volume (0.7 ± 0.1 ml),	99.78 ± 0.92	0.92
1 M NaOH volume (1.3 ± 0.1 ml)	97.22 ± 0.68	0.70
Method II		
pH of 0.2 M BRB solution (4.0 ± 0.1)	99.43 ± 0.54	0.54
1.7 × 10^−4^ M EB volume (0.6 ± 0.1 ml)	96.71 ± 0.51	0.53

#### 3.3.6. Specificity

Specificity for both methods was confirmed by determining ATX in its pharmaceutical dosage forms and table 7 showed good % was found without any interference from the excipients and additives in ATX capsules, which confirmed the good specificity of both methods.

**Table 7.** Assay results for the determination of ATX in commercial capsules.

parameter	Method I		Method II		comparison method [6]
	conc. taken (µg ml^−1^)	% found^a^	conc. taken (µg ml^−1^)	% found^a^	% found^a^
Atomox Apex ® capsules (40.0 mg ATX/cap)	0.2	101.69	0.2	102.77	101
	0.4	98.13	0.5	100.21	99.9
	0.8	102.19	1.0	102.25	98.4
	1.2	100.29	2.0	98.67	100.85
	1.6	99.33	3.0	102.59	
mean		99.93		101.29	100.03
s.d.		1.78		1.79	1.19
*t- test*		0.10 (2.36)^b^		0.67 (2.31)^b^	
*F-test*		2.22 (9.01)^b^		3.61 (9.11)^b^	

^a^Each result is the mean of three separate determinations.

^b^The values between brackets tabulated *t*- and *F*- values at *p* = 0.05[34].

### 3.4. Applications

#### 3.4.1. Pharmaceutical dosage form

Both methods were applied for the determination of ATX in Atomox Apex® capsules and the obtained results were compared statistically with the results of the comparison method [6] (table 7) by using Student's *t*-test and variance ratio *F*-test [34]. These results showed that the calculated values were smaller than the tabulated ones, which indicates that there was no significant difference in the accuracy and precision of both methods. The good sensitivity, accuracy and precision of both methods confirms the suitability of these methods for analysis of ATX in its dosage forms.

#### 3.4.2. Content uniformity test

The proposed methods were perfectly suitable for testing content uniformity of ATX in its commercial capsules because of their high sensitivity and ability to determine ATX in single capsule extract with sufficient accuracy and precision. The test steps were performed following USP guidelines [21], where the acceptance value (AV) was calculated and found to be less than the maximum allowed value AV (L1) [21]. These results indicate the excellent uniformity of ATX in its commercial capsules (table 8).

**Table 8.** Results of content uniformity test using the proposed methods.

parameter	capsule no.	percentage of the label claim (Method I)	percentage of the label claim (Method II)
data	1	100.01	97.77
	2	97.81	98.41
	3	99.33	101.29
	4	98.32	102.88
	5	102.73	99.05
	6	99.16	98.73
	7	98.69	99.69
	8	100.08	102.25
	9	99.37	100.97
	10	102.05	101.61
mean		99.75	100.27
s.d.		1.56	1.76
%RSD		1.56	1.75
acceptance value (AV)		3.74	4.22
max. allowed AV (L1) [21]		15.00	15.00

### 3.5. Assessment of greenness of the proposed methods

Recently the greenness of an analytical method has had the same importance as its sensitivity since it protects humans from the harmful effects of chemicals. The analytical method is considered green if it is performed without the use of toxic organic solvents, time-consuming derivatization procedures, hazardous waste production and excessive energy consumption.

There are various techniques to assess the greenness of analytical methods, including: The National Environmental Methods Index (NEMI) [35], analytical eco-scale score [36] and recently the Green Analytical Procedure Index (GAPI) [37].

These assessment methods were applied to the proposed techniques, as in table 9. The NEMI pictogram was carried out and revealed that these methods met all the criteria to be green. The used reagents were not considered persistent, bio accumulative, toxic [38] and hazardous [39] by the EPA's Toxic Release Inventory [40,41], volume of waste was less than 50 g or L and pH value was found to be within the range 2–12. Even though NEMI is time-consuming and provides only qualitative determination, it is a feasible readable technique [35].

**Table 9.** Results for assessment of greenness of the proposed methods.

(1) Green Analytical Procedure Index (GAPI) For both methods	
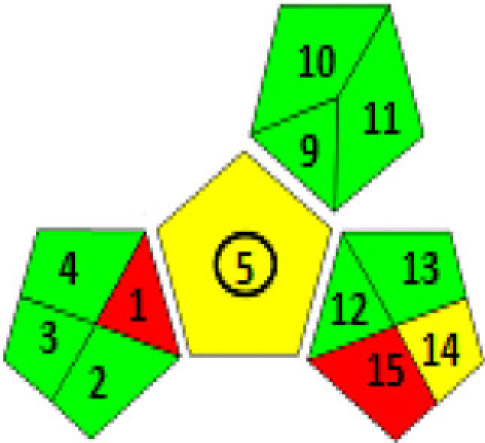	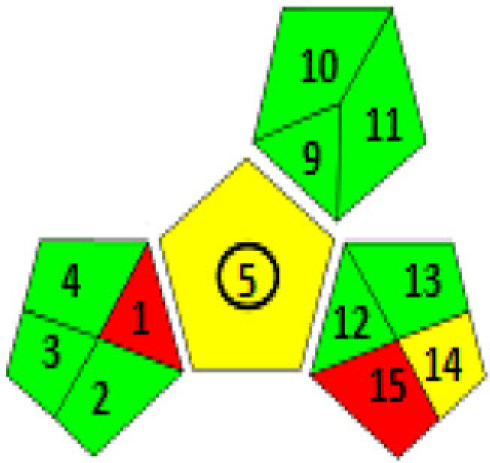
Method I	Method II
(2) NEMI pictogram for both methods	
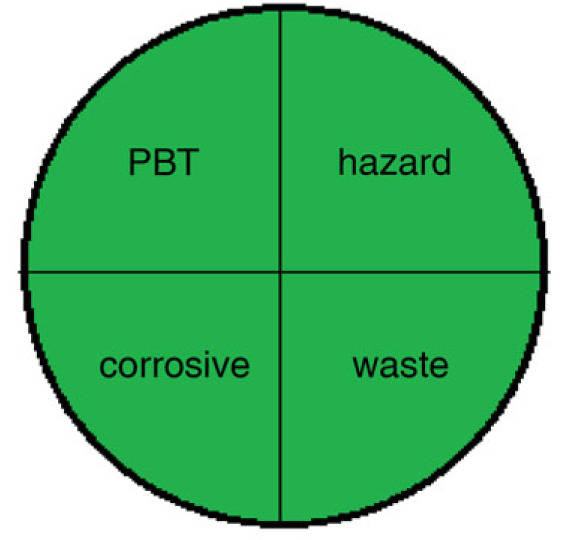	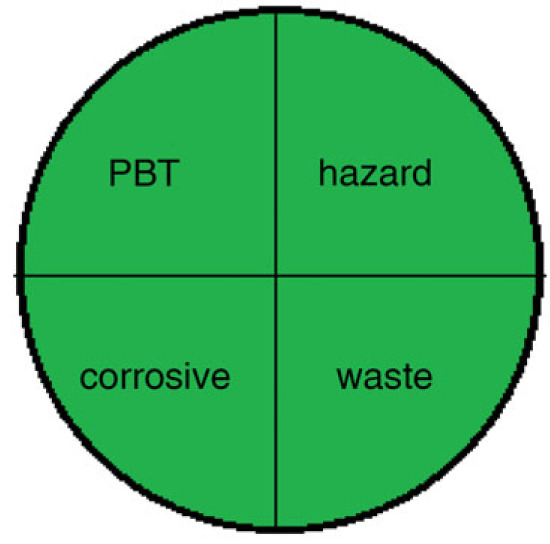
Method I	Method II
(3) analytical eco-scale score for both methods	
for Method I	
item	penalty points
1-reagent	
SDS, 0.7 ml	0
2-spectrofluorimeter	0
3-occupational hazard	0
4-waste	3
total penalty points	3
analytical eco- scale score	97
for Method II	
item	penalty points
1-reagent	
EB, 0.6 ml	1
BRB, 1 ml	2
2-spectrofluorimeter	0
3-occupational hazard	0
4-waste	3
total penalty points	6
analytical eco-scale score	94

Analytical eco-scale is a semi-quantitative tool which relies on various factors including the hazardousness and amount of reagents, waste production and method of treatment, and energy consumed. It requires that the total penalty points are calculated and subtracted from 100 (reference value) and the nearer the value to 100, the greener the analytical method. In these suggested techniques, the score was 97 for Method I and 94 for Method II, confirming the excellent greenness of these approaches [36].

In addition, a new technique was recently developed for evaluating the greenness [GAPI]. It also applies a pictogram to assess the environmental effect of each stage of an analytical method by the use of colour scale of three levels: green, yellow and red, indicating low, medium and high environmental effect. [37].

Table 9 illustrates that the major criteria of GAPI were fulfilled in these proposed techniques except for fields 1,15 (red) which related to the off-line sampling and no treatment of the waste, respectively. And fields 5,14 were coloured yellow because of carrying out the sampling procedure and the formation of 10 ml waste per sample, respectively. Despite this, GAPI was applied for the estimation of ATX in their capsules; the outcomes were coloured yellow, indicating simple preparation (filtration) and the usage of green solvent (distilled water). Overall, these results indicated the greenness of the proposed techniques and their safety for humans and the environment.

## 4. Conclusion

Two green, simple and economic spectrofluorimetric methods were described and validated as per the ICH Guidelines [33] for sensitive determination of ATX in its pure and commercial capsules. These methods involved simple measurement of either the enhanced native fluorescence of ATX or its quenching effect on the EB native fluorescence.

The suggested methods are accurate, specific and highly sensitive for application to the assay of ATX in its capsules and to verify the content uniformity of capsules. Furthermore, neither method required complicated handling associated with other techniques such as HPLC or capillary electrophoresis and did not use hazardous organic solvents, which means they are time saving, cost effective and have a lower impact on the environment. In addition, the assay used a simple fluorimetric apparatus which made the presented techniques perfectly suitable for routine quality control in pharmaceutical manufacturing based on their greenness, high sensitivity and selectivity.

## Data Availability

The data are available from the Dryad Digital Repository: https://doi.org/10.5061/dryad.280gb5mt4 [42].
